# Tunable order–disorder continuum in protein–DNA interactions

**DOI:** 10.1093/nar/gky732

**Published:** 2018-08-11

**Authors:** Sneha Munshi, Soundhararajan Gopi, Gitanjali Asampille, Sandhyaa Subramanian, Luis A Campos, Hanudatta S Atreya, Athi N Naganathan

**Affiliations:** 1 Department of Biotechnology, Bhupat and Jyoti Mehta School of Biosciences, Indian Institute of Technology Madras, Chennai 600036, India; 2 NMR Research Centre, Indian Institute of Science, Bangalore 560012, India; 3 National Biotechnology Center, Consejo Superior de Investigaciones Científicas, Darwin 3, Campus de Cantoblanco, 28049 Madrid, Spain

## Abstract

DNA-binding protein domains (DBDs) sample diverse conformations in equilibrium facilitating the search and recognition of specific sites on DNA over millions of energetically degenerate competing sites. We hypothesize that DBDs have co-evolved to sense and exploit the strong electric potential from the array of negatively charged phosphate groups on DNA. We test our hypothesis by employing the intrinsically disordered DBD of cytidine repressor (CytR) as a model system. CytR displays a graded increase in structure, stability and folding rate on increasing the osmolarity of the solution that mimics the non-specific screening by DNA phosphates. Electrostatic calculations and an Ising-like statistical mechanical model predict that CytR exhibits features of an electric potential sensor modulating its dimensions and landscape in a unique distance-dependent manner, while DNA plays the role of a non-specific macromolecular chaperone. Accordingly, CytR binds its natural half-site faster than the diffusion-controlled limit and even random DNA conforming to an electrostatic-steering binding mechanism. Our work unravels for the first time the synergistic features of a natural electrostatic potential sensor, a novel binding mechanism driven by electrostatic frustration and disorder, and the role of DNA in promoting distance-dependent protein structural transitions critical for switching between specific and non-specific DNA-binding modes.

## INTRODUCTION

DNA-binding protein domains (DBDs) are structurally and conformationally distinct from their counterparts that bind proteins. This arises from two specific factors: the large negative charge density on DNA and the necessity to scan millions of very similar sequences to find their target site and promote regulation (1–3). The negative charge density from the phosphates on DNA requires the DBDs to possess equal but positively charged residues in close vicinity in their 3D structure to promote efficient binding (‘electrostatic complementarity’ (4)). This co-evolved protein structural feature in turn leads to large frustration (5), a phenomenon that is also observed in the active sites of many proteins and enzymes (5–7). The energetic or electrostatic frustration helps the DBDs to sample structurally different conformations in equilibrium as the unfavorable interactions just about keeps the protein folded. Some conformations possess the right orientation of charged residues for specific and tight binding, while the other conformations promote non-specific and weak binding. This inherent plasticity enables DBDs to quickly explore numerous and energetically degenerate sequences with ease (8–11).

The extent of specific and non-specific interactions between DBDs and DNA is in turn determined by the protein primary sequence, the folded structure and even by the folding mechanism. This can lead to several interesting folding–binding–translocation–regulation mechanisms—folding-rate acceleration (12), folding-upon-binding/binding-upon-folding (13,14) or combination of the two (15), synergistic folding (14,16,17), ‘fly-casting’ (18,19), ‘monkey-bar’ binding (20), conformational switching (21–26), homo- versus heterodimerization (27) and protein co-localization (28–30)—all of which determine the extent of time the protein spends bound to DNA, its 1D diffusion coefficient relative to the time spent freely diffusing in solution (14,31–34) and hence fine-tuned expression of genes. In many cases, the differences between specific and non-specific binding poses are subtle (8,11,22,35) and can result in distinct cooperative effects (21,36).

One underlying theme to all of the mechanisms reported above is the conformational malleability of DBDs. The co-evolved but frustrated landscape of DBDs can thus manifest as complex binding thermodynamics (37–40), anomalous heat capacity profiles (41), downhill-like folding mechanistic behaviors (14) and large dynamics even in the DNA-bound form (42,43). An extreme case is that of a DBD folding upon binding to DNA (or vice versa), while it remains disordered in the absence of DNA. Such a phenomenon is frequently observed in protein–protein interactions where one partner remains folded while the other protein domain folds upon binding (44,45). Cytidine repressor (CytR) DBD (referred to as CytR) is an intrinsically disordered protein (IDP) that binds its target *udp* half-site with a weak affinity (46), and promiscuously to multiple other sites (47), despite assuming a folded-like structure on binding. In fact, a detailed analysis revealed that CytR is highly frustrated electrostatically, samples multiple conformations in equilibrium driven by non-specific and a continuous collapse transition, but this conformational heterogeneity is also translated into binding heterogeneity (36), reminiscent of the ‘fuzzy’ complexes in protein–protein interactions (48).

In the case of fully folded proteins or ligands that carry excess positive charges, the large negative electrostatic potential of DNA is expected to merely ‘pull’ them toward the center of attraction thus promoting binding. However, what happens when the protein is less structured or disordered, as is the case for CytR? If they are frustrated electrostatically due to the presence of excess positive charges, the intuitive expectation is that the non-specific electrostatic potential should promote folding of such disordered proteins in a distance-dependent manner due to progressively stronger charge screening as the protein approaches DNA. We test this hypothesis in the current work through salt-screening experiments on CytR and a statistical mechanical model. We identify a unique mechanism determining the heterogeneous binding of CytR to DNA, a feature that could also be prevalent in other disordered and even folded proteins.

## MATERIALS AND METHODS

### Protein expression, purification and spectroscopy

The protocol for overexpression of CytR, purification and spectroscopic measurements is outlined in detail in (36). The 2D [^15^N, ^1^H]-HSQC spectra were recorded at 298 K on a Bruker Avance III 800 MHz nuclear magnetic resonance (NMR) spectrometer equipped with a cryogenically cooled triple resonance probe. The spectra were acquired with 4 transients and 256 complex points, with an average measurement time of 20 minutes at protein concentrations of 200–400 μM.

### Analytical ultracentrifugation (AUC)

Sedimentation velocity experiments were carried out in Optima XL-I (ultraviolet (UV)–VIS absorbance at 280 nm and interference detection) at 48 000 rpm and at 298 K at a CytR concentration of ∼50 μM. The sedimentation velocity profiles were collected at different time intervals and the sedimentation coefficients calculated using SEDFIT with corrected buffer densities and viscosities.

### Kinetics

Ionic strength and urea-dependent kinetic experiments were performed at 298 K, pH 7.0 in a Chirascan SF.3 Stopped Flow instrument (Applied Photophysics Ltd.; dead-time ∼2 ms) coupled to a thermostated water bath. The sole tyrosine in CytR (Y53) was excited at 280 nm, kinetic traces collected, averaged (from at least six repeats with one-minute equilibration between individual repeats) and fit to single-exponential functions. The starting buffer for ionic strength dependent folding and unfolding experiments was at 11 and 2500 mM ionic strength, respectively. The starting protein concentrations were ∼200 μM with the final concentrations (after mixing) of ∼18 μM.

The kinetics of association was monitored by recording anisotropy traces of Alexa-532 labeled *udp* half-site with excitation and emission wavelengths of 530 and 570 nm, respectively. Both DNA and CytR were dissolved in 50 mM sodium phosphate buffer, 30 mM sodium chloride and 1 mM ethylenediaminetetraacetic acid, pH 6.0. Binding was initiated by 1:1 mixing of Alexa-532 labeled DNA with excess CytR DBD mimicking pseudo-first order conditions. For each protein concentration, six traces were recorded at an interval of one minute and averaged.

### Differential scanning calorimetry (DSC) and variable-barrier (VB) model

The scanning calorimetry experiments were recorded in a MicroCal VP-Capillary DSC with an automated sample injector as described before (36). The variable-barrier (VB) model analysis was performed on the absolute heat capacity thermograms of CytR at the three explored ionic strength conditions by fixing the Freire folded baseline (49). The final parameters at [43, 600 and 1300] mM ionic strength conditions are: Σ*α* = [1554.9, 585.1 and 121.1] kJ mol^−1^; *T*_0_ = [291.7, 313.6 and 337.3] K; *β* = [−173.7, −12.14 and 0.12] kJ mol^−1^; *f* = [0.535, 0.631 and 0.903].

### Wako–Saitô–Muñoz–Eaton (WSME) model

The Wako–Saitô–Muñoz–Eaton (WSME) model (50,51) in its latest version includes contributions from intramolecular van der Waals interactions, electrostatics and solvation apart from conformational entropy (52,53) (see Supporting Methods). In the current work, the basic WSME model terms of CytR DBD (PDB ID: 2L8N) are supplemented with an extra weighting term on residue *j*.

\begin{equation*}{w_i} = \mathop \prod \limits_{j = 1}^N \exp \left( { - \Delta {G_{j,{\rm DNA}}}\ {\rho _j}/RT} \right),\end{equation*}

where *ρ_j_* is the folded status of the residue *j* in microstate *i, N* is the number of residues, *R* is 8.314 J mol^−1^ K^−1^ and *T* is the temperature. The free energy contribution due to the interaction of residue *j* with DNA (*ΔG_j_,*_DNA_) includes van der Waals interactions as identified using a Gō-like approach employing a 5 Å heavy atom distance cutoff and electrostatic interactions between the residue *j* of CytR and every phosphate group on DNA (non-specific charge–charge interactions). The bound conformation of CytR was modeled in PyMOL (54) with the LacR structure (1CJG) as the template and energy minimized in GROMACS (55). A series of poses spatially displaced from DNA were generated in PyMOL and used for model predictions on the role of DNA. The calculation of overall partition function, free energy profiles and residue probabilities are described in detail elsewhere (50,52). The final model parameters are—mean interaction energy per native contact (*ξ*) = −217.1 J mol^−1^, entropic cost for fixing a residue in native conformation (Δ*S*_conf_) = −33.31 J mol^−1^ K^−1^ per residue and the temperature independent heat capacity per native contact ($\Delta C_{\rm p}^{{\rm cont}}$) = −2.33 J mol^−1^ K^−1^. An uniform dielectric constant (*ϵ* = 29) was used to scale both intramolecular (*ϵ*_prot_; between the charged residues on the protein (52,56)) and intermolecular (*ϵ*_prot,DNA_; between protein and DNA) electrostatic interactions. The latter was also varied from the 29 to 74.3 with little overall changes in the model predictions.

### Electrostatic calculations

The net electrostatic interaction energy (pH 7.0, 310 K, 100 mM ionic strength) between folded CytR and DNA was calculated at different distances employing a simplified Debye–Hückel formalism (52). Tanford–Kirkwood (TK) electrostatic calculations were carried out as before (57,58) to extract the pair-wise charge–charge interaction energies of CytR at varying ionic strength conditions. The electrostatic potential around CytR and DNA was calculated with the Adaptive Poisson−Boltzmann Solver (59,60). The charges were assigned using the PDB2PQR module employing AMBER charge set, while the residue protonation states at pH 7 were assigned using the PROPKA routine. The non-linear PB equations were numerically solved at 310 K, on a 193 × 193 × 193 Å grid with 100 grid points per Å^2^ for surface construction. The dielectric constant was set to default (78.5 for solvent and 2.0 for protein interior) and the ion radii were set to 2 Å. The net electrostatic potential of the molecules was calculated at 2 Å from the molecular surface.

## RESULTS AND DISCUSSION

### Electrostatic frustration in CytR

**Figure 1.**
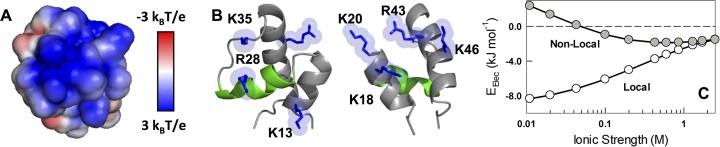
Electrostatic frustration in CytR (**A**). The large positive surface potential on the DNA-binding face. (**B**) Left: Same orientation as in panel (A) but with the residues labeled. Right: Identity of the positively charged residues that do not bind DNA but are still frustrated. (**C**) Charge–charge interaction energy of folded CytR as a function of ionic strength and as calculated from the TK algorithm. Non-local interactions are identified with a sequence separation >4.

Unfavorable electrostatic interaction is a characteristic feature of DBDs that bind DNA. In this regard, the folded CytR (i.e. the structure in the presence of DNA) exhibits a unique feature wherein specific residues that are far from DNA (K18, K20, R43, K46) are as frustrated, if not more, as those residues that come together to form favorable interactions with the DNA backbone (K13, R28 and K35; Figure 1A and B). TK electrostatic interaction energy calculations (57,58) reveal that CytR exhibits unfavorable interactions between helices 1 and 3, i.e. long-range interactions that hold the protein together.

This can be observed in the plots of the electrostatic interaction energy as a function of ionic strength: increasing ionic strength screens local interactions making them more unfavorable while promoting long-range (non-local) charge–charge interactions (Figure 1C). Beyond 500 mM ionic strength, both the interactions contribute equally to the overall stability. Electrostatic potential calculations in fact indicate that as the ionic strength is increased, the unfavorable interactions are progressively screened thus reducing electrostatic frustration (Supplementary Figure S1). These observations hint that an intrinsic conflict between local and non-local electrostatic interactions could be one of the fundamental factors contributing to the low stability and structure of CytR.

### Charge screening promotes structure in CytR

**Figure 2.**
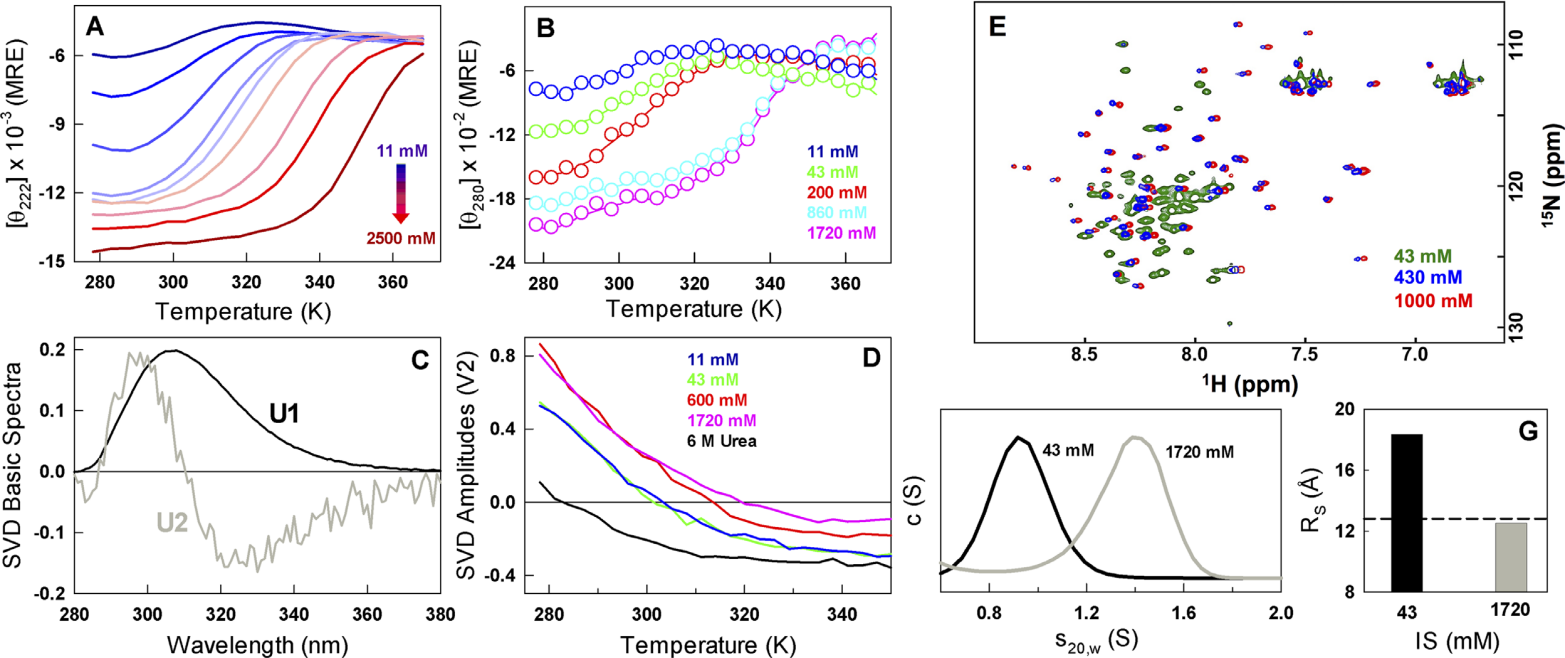
Charge screening promotes a continuum of structural states in CytR (**A** and **B**). Far-UV and near-UV CD unfolding curves at varying ionic strength conditions. MRE represents mean residue ellipticity in units of deg. cm^2^ dmol^−1^. (**C** and **D**) Global singular value decomposition (SVD) of temperature–wavelength fluorescence data of CytR unfolding at different ionic strength conditions. The first and second basis spectra (which shows a red shift) are displayed in black and gray, respectively. The amplitudes of the second basic spectra as a function of temperature are shown in panel (D) highlighting the temperatures at which they change sign depending on the stability conditions. (**E**) Overlay of ^15^N,^1^H-HSQC spectra at 43 mM (green), 430 mM (blue) and 1000 mM (red) ionic strength conditions. (**F** and **G**) Corrected sedimentation velocity distributions and the apparent Stokes radii (*R*_S_) of CytR at the two extreme ionic strength conditions. The dashed horizontal line in panel (G) signals the dimensions of a perfect sphere.

To explore the predictions experimentally, we systematically increase the ionic strength of the buffer by adding salt and probe for the effect on secondary and tertiary structure of the protein. The far-UV CD monitored secondary structure increases continuously on adding salt ranging from −6000 deg. cm^2^ dmol^−1^ at 298 K and 11 mM ionic strength buffer to −15 000 deg. cm^2^ dmol^−1^ at 2.5 M ionic strength conditions (Figure 2A). In fact, the signal at 298 K approaches that of the folded PurR or LacR (which are fully folded in the absence of DNA) at the highest salt concentrations indicating a fully folded domain. The apparent melting temperature also shows an increasing trend ranging from ∼303 K at 11 mM, ∼340 K at 1.7 M and finally to 345 K at 2.5 M ionic strength conditions (Figure 2A). A shorter CytR construct, which does not include the long unstructured N- and C-terminal residues, exhibits a similar increase in structure and stability highlighting that the stability modulation is an intrinsic feature of the sequence region that folds in the presence of DNA (Supplementary Figure S2).

CytR possesses a single tyrosine (Y53) thus allowing us to probe for the changes in the tertiary environment on ionic strength modulation. The near-UV CD spectral magnitude of CytR increases with salt and shifts to the right mirroring far-UV CD observations providing a clear indication that the overall tertiary structure increases concomitantly with secondary structure (Figure 2B). The fluorescence emission of tyrosine exhibits a small red-shift on increasing temperatures providing an alternate probe to monitor structural changes. This component of the spectra can be extracted by performing singular value decomposition of the global temperature–wavelength–ionic strength data (Figure 2C) (36). Such an analysis reveals that the red-shift dominates the observed spectrum at ∼283 K in the presence of urea wherein the protein is fully unfolded, while it increases to ∼301 K at 43 mM ionic strength and eventually to just ∼319 K at 1.7 M ionic strength (Figure 2D). The large difference in the apparent inflection points at 1.7 M (∼22 K comparing against far- or near-UV CD under identical conditions) indicates that though the protein gains structure with increasing ionic strength, the changes are decoupled thermodynamically.

At low ionic strength, the ^1^H-^15^N HSQC peaks of CytR are cluttered indicating heterogeneity in chemical environment (green in Figure 2E) that decreases with increasing ionic strength (blue and red in Figure 2E and Supplementary Figure S3). At 1 M ionic strength, the peaks are as dispersed as in the NMR spectrum obtained in the presence of DNA (46) indicating that the structural changes encompass the entire protein. The gain in structure and stability is intuitively expected to go hand-in-hand with a compaction of the native ensemble at 298 K. AUC experiments accordingly result in relative CytR dimensions (*R*_S_) of 18.4 and 12.5 Å at low and high ionic strength conditions, respectively (Figure 2E and F). The dimensions of CytR at high salt are as compact as that expected for a perfect sphere, i.e. 12.8 Å, suggestive of a well-folded protein domain.

### Evidence for continuous structural acquisition

**Figure 3.**
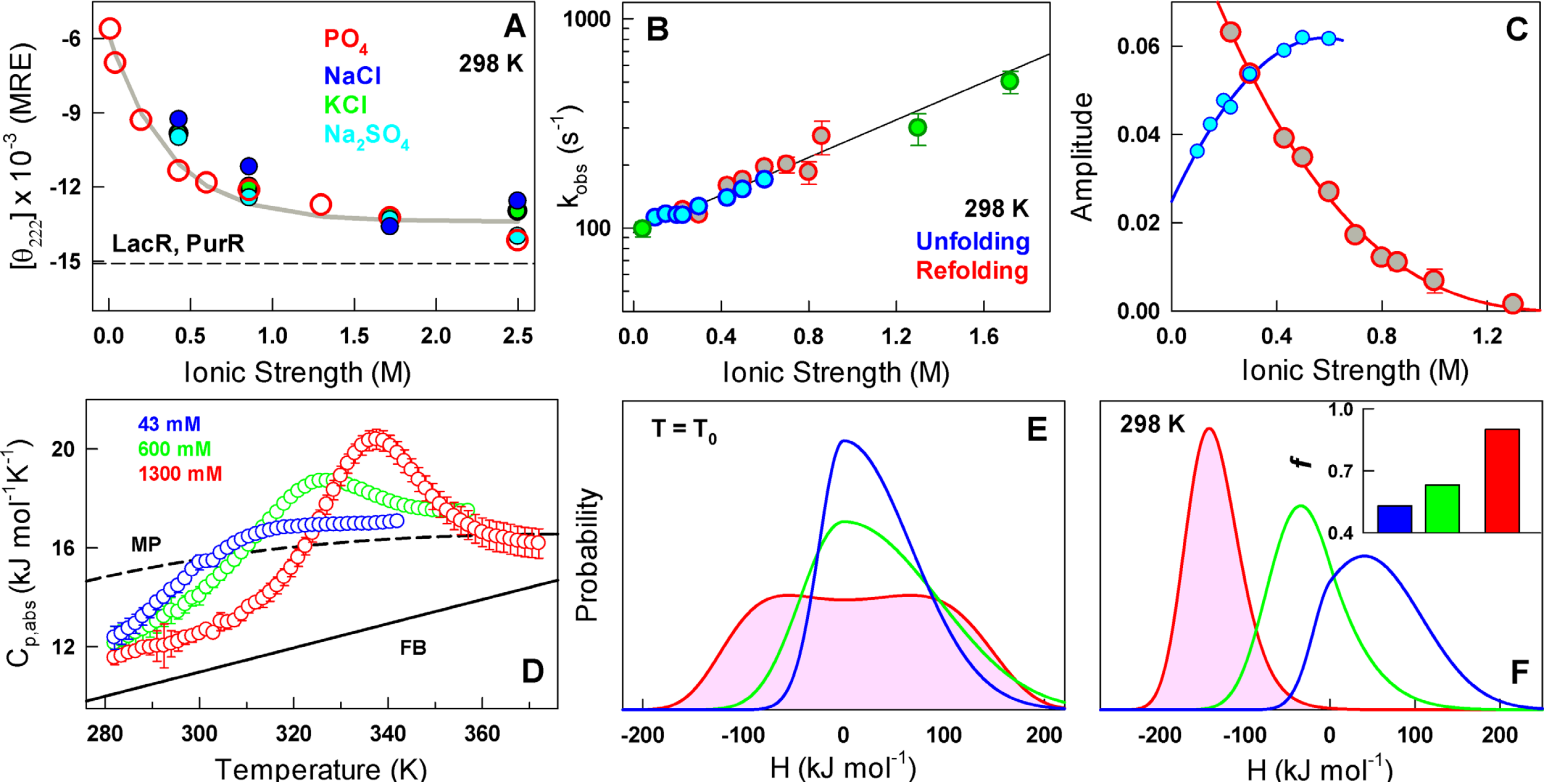
Evidence for a continuous structural acquisition (**A**). Non-specific charge screening promotes structural gain in CytR as monitored by far-UV CD at 222 nm with various ions. (**B**) Observed relaxation rates from stopped-flow experiments in the folding (red), unfolding (blue) direction and from extrapolation of rates estimated from urea-induced changes in equilibrium at 43, 1300 and 1720 mM ionic strength conditions (green). (**C**) The amplitudes following the color-code in panel (B). (**D**) Absolute heat capacity profiles of CytR at the specified ionic strength conditions. FB and MP stand for the Freire and Makhatadze–Privalov unfolded baselines, respectively. (**E** and **F**) Probability densities at *T*_0_ and 298 K from a VB model analysis of the heat capacity profiles following the color code in panel (D). Inset to panel (F): asymmetry factor, a measure of structural compactness or the native ensemble width, at different ionic strength conditions.

The gain in CytR structure is also found to be independent of the nature of salt added thus ruling out specific binding (Figure 3A). The range of salt concentrations required to promote structural transitions in CytR are 2–3 orders of magnitude more than that reported in Ribonuclease P protein that folds in the presence of salt. Ribonuclease P protein folds in a cooperative manner on increasing the phosphate or sulphate concentration in buffer from 0 M to a mere 20 mM suggestive of specific binding (61). The larger range of salt concentrations required to induce structural changes in CytR is evidence that the stabilization mechanism is most likely driven by non-specific electrostatic screening of unfavorable charge–charge interactions within the protein (Figure 1), similar to observations in acid-denatured BBL (62,63). The question then is: does salt stabilize folded or binding-competent conformations over disordered states in a two-state-like manner or does it contribute to a continuous stabilization of folded-like conformations, akin to a continuous or second-order transition (64)?

Chevron-like kinetic behaviors are typical of two-state systems; in other words, upon denaturant addition the observed rate constants decrease, reach a minimum and increase again (65). This arises from the fact that at the midpoint of unfolding transition the rate determining barrier height is maximal in a two-state-like system, while decreasing on either side of it. In the case of CytR, since the ionic strength conditions can be modulated to tune the relative stability of states, the corresponding relaxation rates should be chevron-like if the transition is over a large free-energy barrier. Ionic strength-modulated kinetics of CytR surprisingly reveals a continuous increase in relaxation rates with increasing structure and stability: the rates range from ∼100 s^−1^ at 43 mM to nearly 500 s^−1^ at 1.7 M with no evidence for a chevron-like behavior (Figure 3B and C). Moreover, a roll-over in rates is not evident near the apparent chemical denaturation midpoint on perturbation with urea at 1.3 and 1.72 M ionic strength conditions, providing additional evidence for a non-two-state transition (Supplementary Figure S4). The slow relaxation rates even at low ionic strength conditions potentially arise from a highly frustrated (or rough) landscape. The rates increase would therefore be a manifestation of reduced electrostatic frustration (i.e. smoother landscape) at higher IS conditions.

The observations above hint that the protein folds in a continuous manner with increasing ionic strength conditions. An avenue to test this expectation is to perform scanning calorimetry experiments at varying ionic strength conditions or extent of charge screening and model the underlying the distribution of states through statistical approaches (49,66). The absolute heat capacity profiles show dramatic differences in the presence/absence of pre-transition baselines and excess heat capacity and are incompatible with the expectation from a two-state-model (Figure 3D and Supplementary Figure S5). There is a large difference between the measured absolute heat capacity at the lowest temperature and the expectation for a folded domain (Freire baseline) at even 1.3 M ionic strength conditions, highlighting the presence of significant residual enthalpic fluctuations.

To quantify the differences, we fit the heat capacity profiles to the VB model of Muñoz and Sanchez-Ruiz (49) that provides estimates of thermodynamic barrier heights and conformational widths of the native ensembles. At the apparent midpoint temperatures (*T*_0_), the probability densities of CytR are unimodal at 43 and 600 mM ionic strength (i.e. one-state-like folding with thermodynamic barrier ≤0), while exhibiting signs of a small thermodynamic barrier (∼0.1 kJ mol^−1^) at 1300 mM ionic strength conditions (Figure 3E). The small barrier appears to coarsely separate the folded-like and unfolded-like conformations (a broad free energy well) and is consistent with the progressively better two-state model fits to the heat capacity profile (but with crossing baselines; Supplementary Figure S5) and the appearance of the sharper excess heat capacity with increasing salt. The probability densities at 298 K are expectedly unimodal at the three ionic strength conditions, with extracted sharpness of the native probability distribution (quantified by the asymmetry factor *f*) increasing from 0.53 at 43 mM, 0.63 at 600 mM to 0.90 at 1300 mM indicative of a more compact ensemble at high ionic strength conditions (inset to Figure 3F). In other words, salt is predicted to progressively or continuously fold CytR with distinct ensembles at different stabilization conditions akin to a one-state system (67) explaining why the folding kinetics does not exhibit a chevron-like behavior. The maximum of the distribution along the order parameter carries additional information on the nature of the ensemble populated; the mode moves from a positive value at 43 mM to successively lower enthalpy values at 600 and 1300 mM (Figure 3F). The conformational behavior of CytR therefore smoothly shifts from disordered and high enthalpy ensemble to molten-globule-like to a low enthalpy compact ensemble at 298 K but still exhibiting downhill features by mere modulation of salt concentration in the buffer.

### The electrostatic potential of DNA and its chaperone-like role

**Figure 4.**
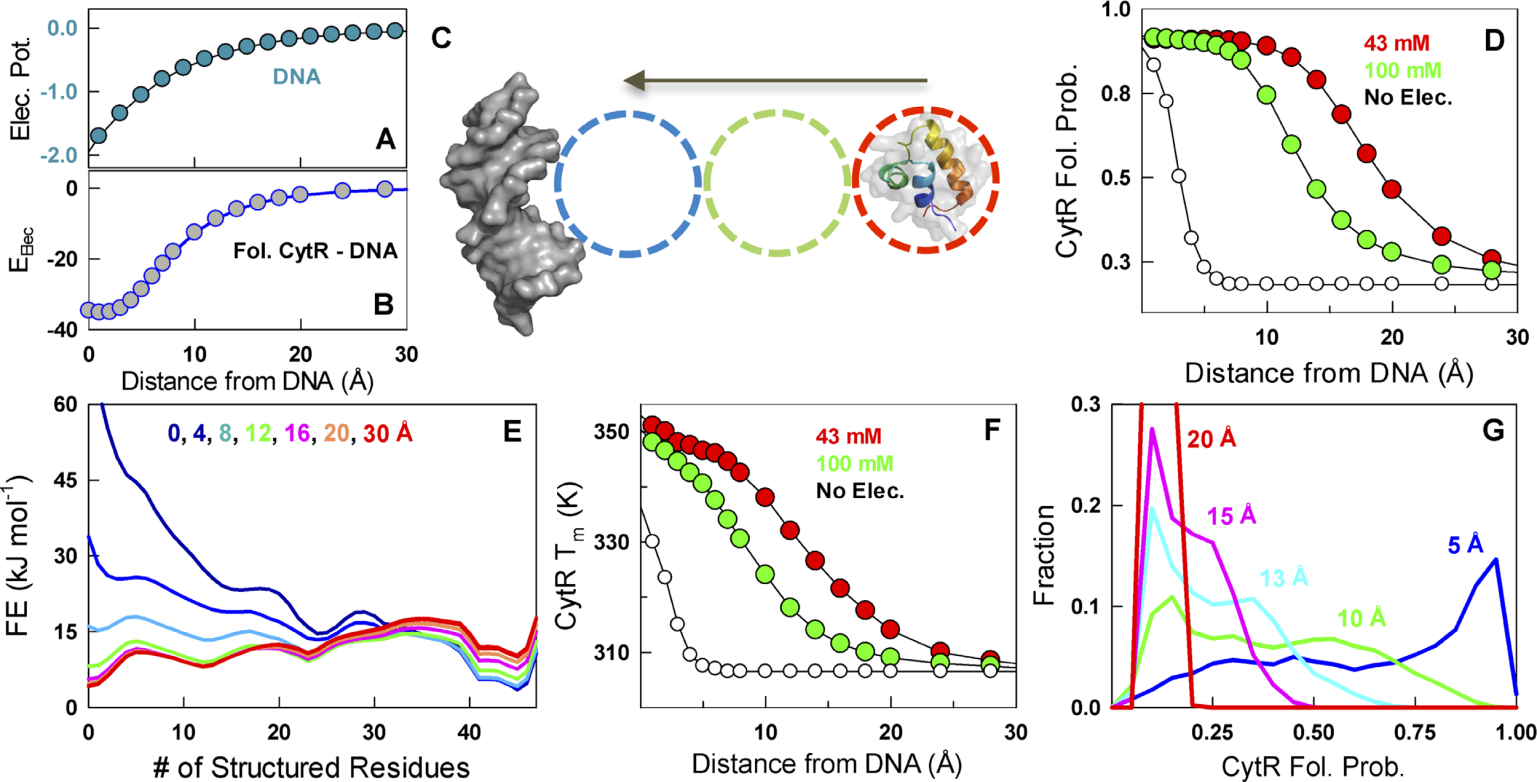
The electrostatic potential of DNA and its chaperone-like role. In all calculations, a distance of zero represents the DNA-bound CytR conformation. (**A**) The electrostatic potential of B-DNA (in *k*_B_*T/e* units) can be felt up till 20–25 Å from the molecular surface acting as a guiding funnel for charged molecules. (**B**) The electrostatic interaction energy (in kJ mol^−1^) between folded CytR and DNA as a function of distance between the two. (**C**) A schematic of the approach employed to calculate distance-dependent structural stability features of CytR from DNA (dark gray surface) through the WSME model. The most-distal pose (red circle) is assumed to have a melting temperature of 305 K as experimentally identified in the absence of DNA (Figure 2). The WSME model predicts the thermodynamic features as the protein is continuously moved toward DNA (green and blue circles). (**D**) Predicted mean residue folding probabilities of CytR, a measure of global structure, with (filled circles) and without (open circles) intermolecular electrostatic terms as a function of protein–DNA distance. CytR folds only when it is very close to the DNA (∼5 Å or less) in the absence of intermolecular electrostatic terms (open circles), guided purely by intermolecular van der Waals interactions. (**E**) One-dimensional free energy profiles of CytR as a function of number of structured residues at varying distances from DNA. (**F**) Predicted changes in melting temperature of CytR as function of distance from DNA. (**G**) The distribution of folded CytR populations at different distances for numerous relative orientations from >345 000 1D free energy profiles. Note that multiple conformational states of CytR are possible even at a CytR–DNA distance of 5 Å.

The observed continuum of conformational behavior with increased charge screening raises the question of why CytR displays this distinct feature. Classic non-linear Poisson–Boltzmann calculations reveal that the electrostatic potential of even 10–24 base-pair DNA fragments extends to nearly 20–25 Å at 100 mM bulk ionic strength conditions (Figure 4A) (68,69). The interaction energy between the folded conformation of CytR and DNA is also progressively favorable with decreasing distances between the two (Figure 4B). Given the continuous folding of CytR with salt or charge screening, it is tempting to speculate that the disordered CytR folds as it approaches DNA in a distance-dependent manner, funneled by the long-range negative electrostatic potential of DNA.

Direct evidence for this unique mechanism is challenging to explore experimentally as it requires single-molecule methods with precise Angstrom-level control of intermolecular distances while at the same time probing for the degree of foldedness of CytR (three-color single-molecule FRET potentially). However, the non-specific nature of the screening effect indicates that it should be possible to model this behavior. We take recourse to the WSME model (50,51), an ensemble-based statistical mechanical model, wherein the phase space of a protein residue is simply represented as native (binary 1) or non-native (binary 0), allowing for an instantaneous ensemble of 2*^N^* microstates for a *N*-residue protein. We extend the classical WSME model (with intramolecular interactions) to include protein–DNA interactions by re-weighting the statistical weights of those residues that interact with DNA through both van der Waals interactions, and specific (52) and non-specific charge–charge interactions between the protein positive charges and the backbone phosphates of DNA (as obtained from the modeled bound structure, see ‘Materials and Methods’ section). The model is parameterized by merely reproducing the apo-CytR melting temperature of ∼305 K, i.e. when CytR is positioned at large distances from DNA (>30 Å, unbound), thus resulting in a disordered conformational behavior (red circle in Figure 4C). The role of non-specific charge–charge interactions at varying distances is directly calculated by merely moving the protein toward DNA along a specific axis (empty circles Figure 4C). In all cases discussed below, a distance of zero represents the bound conformation (∼6 Å from the DNA surface).

CytR is disordered in the absence of DNA with very little secondary structure (Figure 2). As the protein approaches DNA driven by the strong electrostatic potential, the unfavorable charge–charge interactions are increasingly screened thus promoting a folding transition with the folded probability (*P*_F_) going from 0.3 at >20 Å to ∼0.5 at ∼13–15 Å and finally to 1 when close to DNA even before complete binding (<8–9 Å) (Figure 4D). This is also manifested in the CytR free energy profile that goes from near downhill-like in the unfolded side when far from DNA (>20–25 Å) to molten-globule-like with a flat free energy profile (∼13–15 Å) and eventually to downhill-like toward the folded side in the vicinity of DNA (<8–9 Å) (Figure 4E), exactly as observed in experiments with increased salt screening (Figure 3). The corresponding melting temperatures increase continuously from 305 K when far from DNA to near 350 K when fully bound with a *T_m_* of ∼320 K at intermediate distances, very similar to the higher melting temperatures observed in salt-screening experiments (Figure 4F). On removing the specific and non-specific charge–charge interactions between the protein and DNA, CytR exhibits a transition toward the folded state only at very short distances (<5 Å) indicating pure packing effects between protein and DNA driving folding (black in Figure 4D and F). The overall model predictions are insensitive to the magnitude of the dielectric constant of the intervening medium (Supplementary Figure S6), highlighting the robustness of the folding–binding mechanism.

In the calculation above, we have assumed that the relative orientation of CytR does not change as it diffuses toward DNA. This will not hold true as the protein is free to sample conformations in a plane orthogonal to DNA approach axis, particularly when it is far from DNA. To simulate this expectation, we consider the possible orientations of CytR (with respect to DNA) at spacings of 5° in all the three dimensions resulting in >82 000 potential binding poses far from DNA and >20 000 poses close to DNA. For each pose and at specific distances (5, 10, 13, 15 and 20 Å), 1D free energy profiles are generated for a total of 345 022 1D free energy profiles (see ‘Materials and Methods’ section). The predicted distributions of conformational states at specific distances (Figure 4G) follow the overall trend shown in Figure 4E. It is interesting to note that a large conformational distribution is likely even at 5 Å from DNA, thus hinting at the molecular origins of the heterogeneous binding reported in experiments (36).

### Rapid and non-specific DNA binding

What advantage does this mechanism provide to binding? It is important to note that the proposed mechanism is slightly different from ‘fly-casting’ (wherein a disordered charged segment latches on to the DNA from a distance driving folding (18,19)) and is more akin to the ‘conformational selection’ mechanism of binding (70,71) or the ‘electrostatic steering’ observed in simulations of Ets-DNA binding (72) and experimentally in the classic Barnase–Barstar interactions (73). The only difference is that in the ‘continuous conformational selection’ we infer here, the protein can electrostatically pre-organize itself when it is relatively far from DNA without the need for a disordered protein segment to bind DNA. This pre-organization helps the protein to increasingly sample folded-like and potentially binding competent poses as it approaches DNA enabling rapid binding.

**Figure 5.**
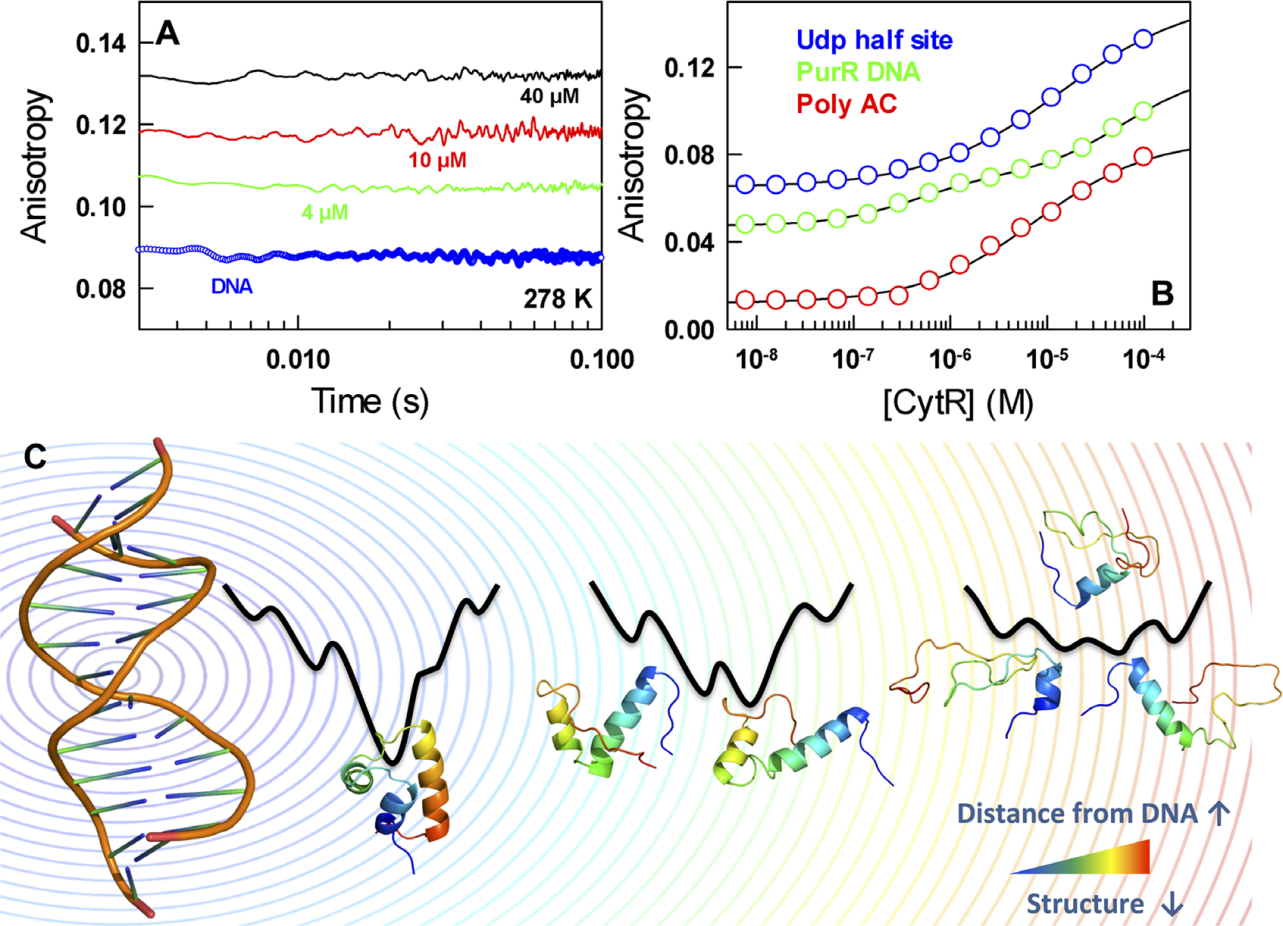
Rapid non-specific binding of CytR to DNA driven by ‘continuous conformational selection’ and electrostatic steering. (**A**) Stopped-flow kinetic anisotropy traces of excess CytR binding to Alexa-532 labeled *udp* half-site (300 nM) mimicking pseudo-first order conditions at 278 K. Note that ‘DNA’ stands for the anisotropy of labeled DNA in the absence of protein (blue), while the other colors represent the kinetic traces at the indicated final protein concentrations. (**B**) Binding isotherms of CytR to different DNA sequences (circles) at 293 K. The data have been shifted vertically for ease of viewing. (**C**) A schematic of the proposed continuous conformational–selection mechanism. CytR is disordered when it is far from DNA due to unfavorable intramolecular interactions. CytR folds continuously as it approaches DNA, i.e. it gains structure and reduces its dimensions, driven by the favorable electrostatic potential of DNA that screens out unfavorable intraprotein charge–charge interactions. At closer distances, the intrinsic conflict between intra- and intermolecular interactions contribute to an increased sampling of structured states thus allowing the protein to rapidly explore both specific- and non-specific binding poses.

In such a mechanism, the binding event should be extremely rapid funneled by large electrostatic forces. In fact, the association between CytR and its native *udp* complement is faster than the dead-time of the stopped flow instrument (∼2 ms) under pseudo-first order conditions even at 278 K wherein the association rate is slow due to increased solvent viscosity (Figure 5A and Supplementary Figure S7). This sets a lower bound on *k*_on_ to be ∼1 × 10^9^ M^−1^ s^−1^ at 278 K, thus being faster than the diffusion controlled limit (10^4^–10^6^ M^−1^ s^−1^) and characteristic of electrostatic steering (73). It is important to note that anisotropy experiments on cMyb (IDP)–KIX (ordered protein) complex formation reveal distinct binding kinetic phases despite exhibiting equilibrium dissociation constants of 1–10 μM (74), very similar to CytR–DNA complex at 293 K (blue in Figure 5B, *K*_1/2_ ∼10 μM from inflection point analysis) (36); the comparison highlights that CytR also dissociates rapidly from DNA contributing to the low binding affinity (36). The second expectation from the proposed mechanism is that such non-specific electrostatic forces should also enable CytR to bind random DNA sequences. True to this, CytR binds random DNA with a similar affinity to the *udp* half-site (*K*_1/2_ ∼10 μM; red in Figure 5B) and even the PurR complement with two overlapping titration profiles (*K*_1/2,1_ ∼0.4 μM and *K*_1/2,2_ > 20 μM; green in Figure 5B) suggestive of two different binding modes (Figure 5B and Supplementary Figure S7). Taken together, our results confirm that the large electrostatic potential of DNA drives the binding of CytR in a non-specific manner.

It is well established that DBDs need to switch conformations in going from non-specific to specific binding modes. What has not been clear is the source of activation energy for such a process that determines the relative populations and interconversion rates to enable efficient balance between 1D sliding and 3D hopping modes. Our experiments and calculations on CytR hint at an answer to this question: the activation energy to drive transitions could be effectively derived from the electrostatic potential of DNA in addition to the random solvent kicks. When CytR is far from DNA, the large electrostatic frustration promotes only unfolded-like conformations (Figure 5C). As it is driven closer to DNA, the free energy landscape of CytR exhibits multiple minima as a result of conflict between destabilizing native interactions and stabilizing long-range protein–DNA interactions; many more structured conformations are thus possible contributing to an efficient sampling of the conformational space (in case of CytR, non-specifically collapsed states would also contribute (36)). Finally, as the protein is very near at the DNA surface, the electrostatic frustration is reduced promoting folded-like conformations albeit with occasional transitions to partially structured states.

## CONCLUSIONS

We effectively find that intramolecular charge screening promotes folded-like conformations in CytR at the expense of unfolded conformations in a purely non-specific manner. The conformational behavior of CytR thus smoothly transitions from being disordered to molten-globule-like to downhill (free energy profile toward the folded well; Figures 2 and 3). This arises from an intrinsic conflict in the charge patterning of CytR with local and non-local effects displaying opposite trends on charge screening (Figure 1). Given that even folded DBDs exhibit a similar and extreme salt-sensitivity (40,75,76), our observations here suggest that such tunable conformational landscape could be a generic feature of DBDs. Statistical mechanical modeling of the interaction behavior highlights the dramatic role of the long-range electrostatic potential of DNA in influencing the conformational landscape and thus the folding of CytR in a uniquely distance-dependent manner (Figure 4). It is important to note that the native conformational landscape of CytR (in the absence of DNA and at 310 K) is close to the collapse transition midpoint (36). The current work therefore highlights that at a certain intermediate distances from DNA, CytR should have access to three macroscopic states—partially structured, unfolded-like and non-specifically collapsed—thus dramatically increasing the conformational space that is sampled.

The ‘continuous conformational selection’ mechanism need not be restricted to protein–DNA interactions, but even to protein–protein or protein–membrane interactions driven by non-specific electrostatic complementarity. In fact, a specific class of proteins termed DNA-mimic proteins exhibits a similar and extensive charge complementary surface as DNA (77). Even ordered protein–DNA and disordered protein–protein interactions (driven by extensive charge complementarity) exhibit distance-dependent trends in terms of their interaction energy (35,78). Our interpretations further highlight the role of DNA and particularly its electrostatic potential in determining the conformational behavior of proteins from a distance. The conformational landscape of CytR is likely to have evolved to specifically fold only when it senses a favorable electrostatic field with the DNA playing the role of a passive macromolecular chaperone. In other words, a ‘folding funnel’ (79) appears only in the vicinity of DNA while being non-existent at other conditions. Such tunable ‘conditional order’ likely enables tight regulation and compartmentalizing functionality to specific regions within the cell.

## Supplementary Material

Supplementary Data
