# Supplementary material for: Tunable order–disorder continuum in protein–DNA interactions
Source: Nucleic Acids Res. 2018 Aug 11;46(17):8700–9. doi: 10.1093/nar/gky732 (PMC6158747; doi:10.1093/nar/gky732)
Supplement: Supplementary Data [file gky732_supplemental_files.pdf]

Supporting Information

Tunable Order-Disorder Continuum in Protein-DNA Interactions

*Sneha Munshi,[1¶] Soundhararajan Gopi,[1¶] Gitanjali A,[2] Sandhyaa Subramanian,[1] Luis A. Campos,[3] Hanudatta S Atreya[2] & Athi N. Naganathan[1]**

[1]Department of Biotechnology, Bhupat & Jyoti Mehta School of Biosciences, Indian Institute of Technology Madras, Chennai 600036, India.

[2]NMR Research Centre, Indian Institute of Science, Bangalore 560012, India.

[3]National Biotechnology Center, Consejo Superior de Investigaciones Científicas, Darwin 3, Campus de Cantoblanco, 28049 Madrid, Spain.

e-mail: athi@iitm.ac.in

Supporting Methods

Wako-Saitô-Muñoz-Eaton Model

The WSME model is a one-dimensional Ising-like statistical-mechanical model that allows for two conformational states for every residue – folded (*1*) and unfolded (*0*) (1,2). A residue level entropic cost (ΔS_{conf}) is introduced to account for the excess number of unfolded-like microstates compared to the folded state. For a *N*-residue protein, this translates to 2^N microstates with the assumption that for any two residues to interact all the intervening residues should be folded. The free energy of each microstate is thus given by,

$$\Delta F = \sum \Delta G_{m,n}^{stab} - T \sum_{m}^{n} \Delta S_{conf}$$

The model energetics in the current version includes contribution from van der Waals interactions (vdW), electrostatics (*elec*) and solvation free energies (*solv*) (3). The effective stabilization free energy of the microstate with a stretch of *n* native residues starting from residue *m* can be described as,

$$\Delta G_{m,n}^{stab} = E_{vdW} + E_{elec} + \Delta G_{solv}$$

The contribution from the van der Waals interactions is estimated using the number of pair-wise heavy-atom contacts from the native PDB structure (i.e. a Gō-like energetics (4)) employing a distance cut-off (r_{cut}),

$$E_{vdW} = \sum_{m,n} \xi_{i,j} \rho$$

where $\rho = 1$ if $r_{ij} \leq r_{cut}$ and $\rho = 0$ otherwise.

Similarly, electrostatic interactions between the charged atoms of residues Arg, Lys, Asp and Glu (at pH 7.0) are modeled by a Debye-Hückel treatment,

$$E_{elec} = \sum_{m,n} K_{Coulomb} \frac{q_i q_j}{\varepsilon_{eff} r_{ij}} \exp\left(-r_{ij} \kappa\right)$$

where K_{Coulomb} is the Coulomb constant (1389 kJ.Å.mol^{-1}), q_i and q_j are the charges on atoms *i* and *j*, respectively, and r_{ij} is the distance between them in the native structure. The effective dielectric constant (ε_{eff}) is fixed to 29 based on previous studies that reproduce the changes in stabilities induced by point mutations of charge residues (5), average thermodynamic behaviors of homologous proteins (3) and the difference in stabilities of mesophile/thermophile protein pairs (5). $1/\kappa$ represents the Debye screening length that accounts for the effects of ionic strength (*I*) and temperature (*T*). The solvation free energy is given by,

$$\Delta G_{solv} = x_{cont}^{m,n} \Delta C_p^{cont} \left[\left(T - T_{ref} \right) - T \ln \left(T / T_{ref} \right) \right]$$

where $x_{cont}^{m,n}$ is the number of native contacts and ΔC_p^{cont} is temperature-independent heat capacity change per native contact at a reference temperature (T_{ref}) of 385K (6).

The total partition function is calculated using the transfer matrix formalism of Wako and Saitô (1),

$$Z(T) = v_l \left[\prod_{i=1}^{N} X_i \right] v_r^{tr}$$

where

$$X_i = \begin{pmatrix} 1 & 1 & 1 & \cdots & 1 & 1 \\ z & 0 & 0 & \cdots & & 0 \\ 0 & H_1^{(i)} z & 0 & & & \vdots \\ & & H_2^{(i)} z & & 0 & 0 \\ & \mathbf{0} & & & H_{N-2}^{(i)} z & H_{N-1}^{(i)} z \end{pmatrix}$$

$$v_l = (1,1,1,\cdots,1)$$

$$v_r = (1,0,0,\cdots,0)$$

and

$$H_k^{(i)} = \exp\left(-\beta \sum_{j=1}^{k} \Delta G_{stab} \right) \qquad (k \le N - i)$$

$$H_k^{(i)} = 0 \qquad\qquad (k > N - i)$$

Here, β *=1/RT* and $z = \exp\left(\Delta S_{conf} \big/ R\right)$ where *R* is 8.314 J mol^{-1} K^{-1}. The folding probability of a residue *j* is calculated from

$$\chi_j = Z^{-1} v_l \left[\prod_{k=1}^{j-1} X_k\right]\left[\frac{\partial X_j}{\partial \ln z}\right]\left[\prod_{k=j+1}^{N} X_k\right] v_r^{tr}$$

The mean folding probability of the residues, $\left\langle \chi_j \right\rangle_T$, is used to generate thermal unfolding curves. The one-dimensional free energy profiles are generated by lumping together partial partition functions of different number of of structured residues (*n*). A detailed explanation of the reweighting approach to model protein-DNA interactions is provided in the main text.

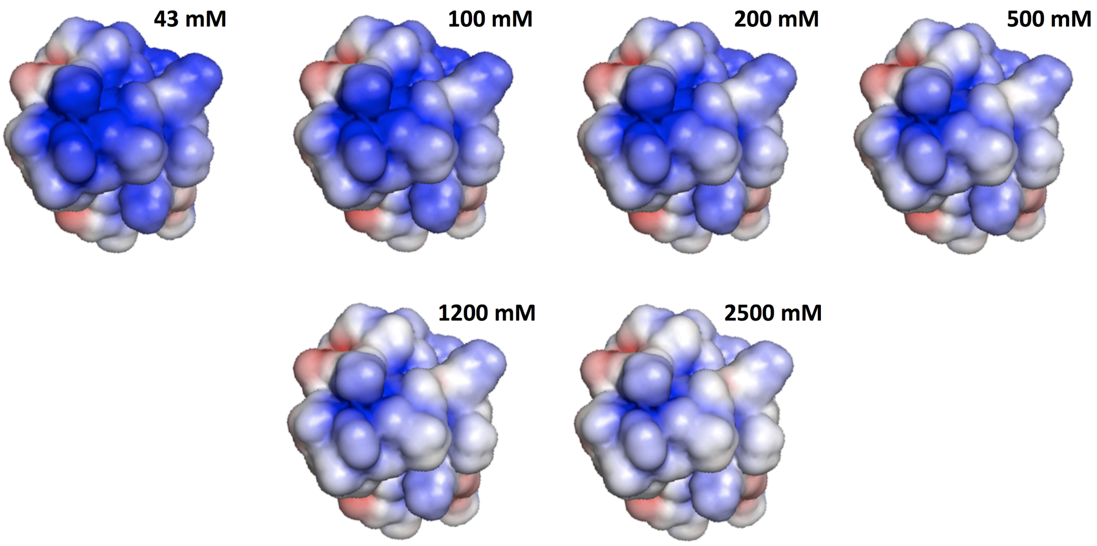

Figure S1. Electrostatic potential surface of the DNA binding face of CytR at varying ionic strength conditions at 310 K and pH 7.0. (Dark blue: 3 $k_B T/e$, White: 0 $k_B T/e$ Dark red: -3 $k_B T/e$)

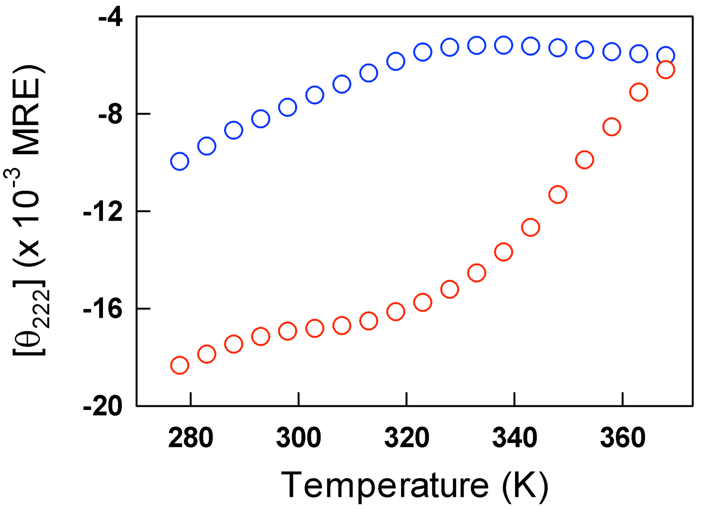

Figure S2. The shorter CytR construct (residues 9-55) displays a similar trend as the WT protein with the extra-unstructured residues at the N- and C-termini (residues 1-66). Blue and red represent the far-UV CD thermal unfolding curves at 43 mM and 2.5 M ionic strength conditions, respectively (MRE, mean residue units of deg. cm^2 $dmol^{-1}$).

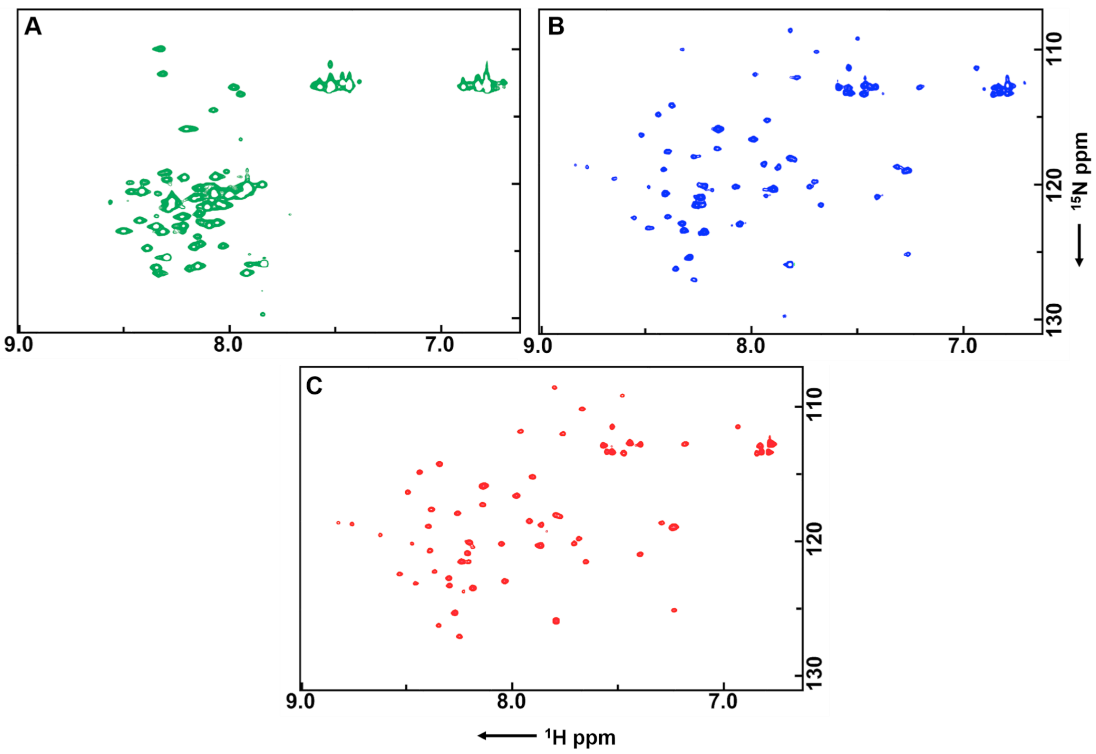

Figure S3. ^1H-^{15}N HSQC spectra of CytR at 43 mM (green), 430 mM (blue) and 1000 mM ionic strength conditions (red).

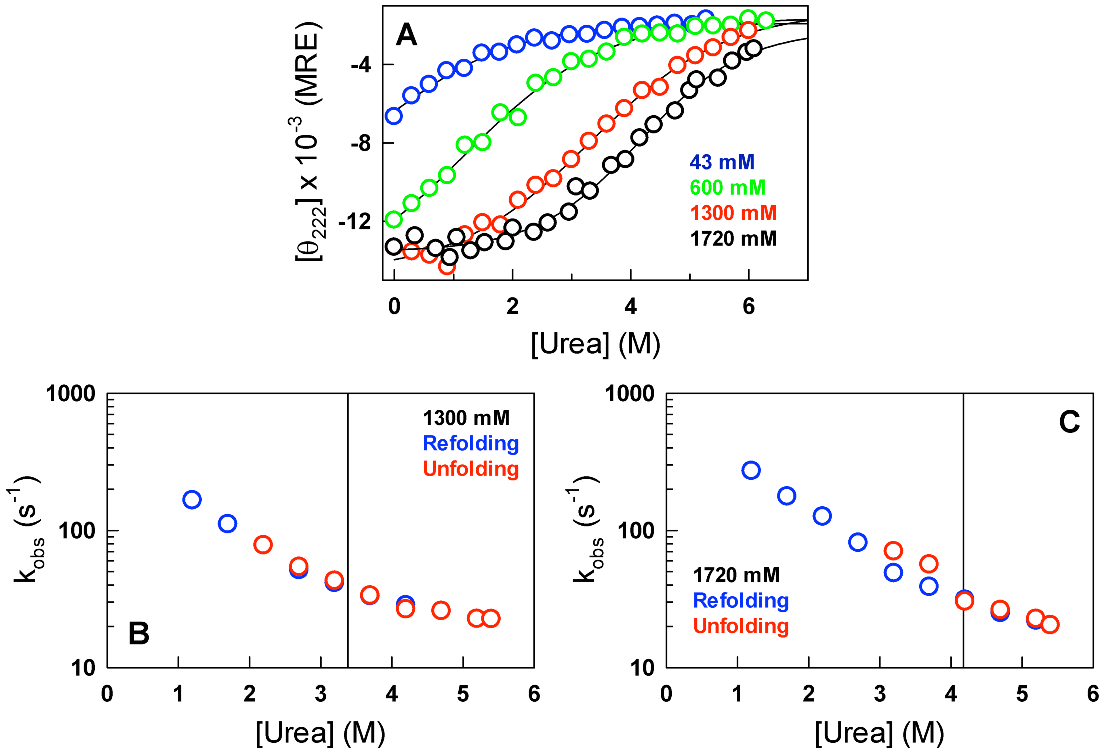

Figure S4. Experiments performed at pH 7.0 and 298 K. (A) Unfolding of CytR as a function of urea at different ionic strength conditions as monitored by far-UV CD at 222 nm. Thin black curves are fits to two-state models to guide the eye and estimate apparent chemical denaturation midpoints (C_m). (B & C) Relaxation rates as a function of urea at the indicated conditions highlighting a non-chevron-like behavior. The vertical lines signal the C_m from equilibrium measurements.

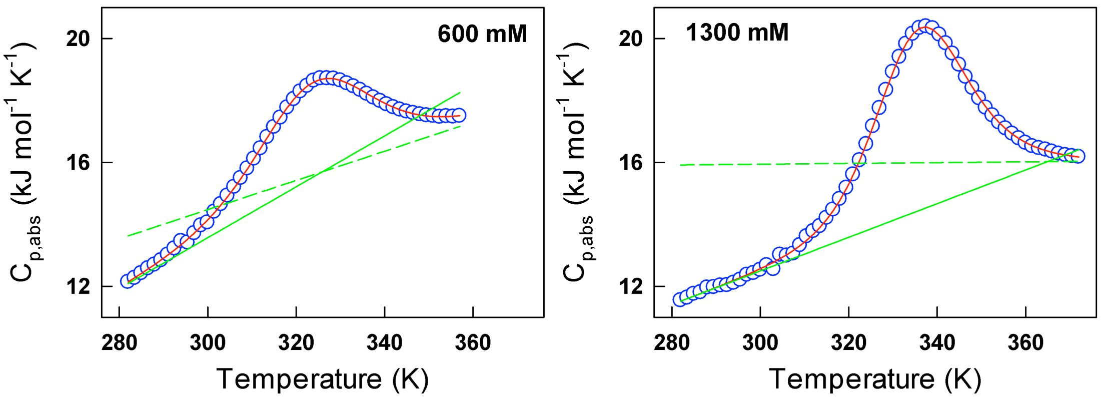

Figure S5. Two-state fits (red) to the scanning calorimetry profiles at the indicated conditions (circles) together with the folded- (continuous green) and unfolded-baselines (dashed green).

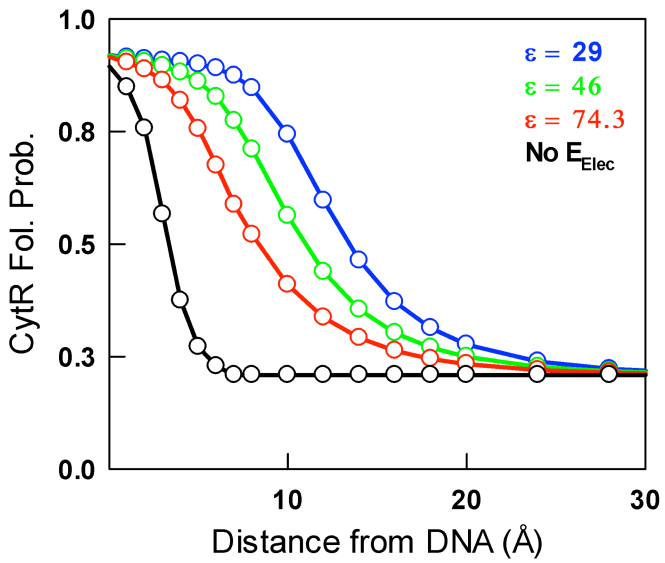

Figure S6. Predicted changes in CytR folding probabilities as a function of distance from DNA at varying dielectric constants of the intervening medium.

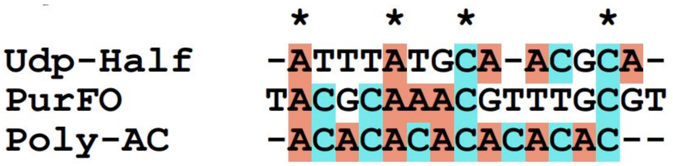

Figure S7 Alignment of the DNA sequences used in this study: natural half-site of CytR (Udp-Half), PurR binding site (PurFO) and Poly-AC DNA.

Supporting References

1. Wako, H. and Saito, N. (1978) Statistical Mechanical Theory of Protein Conformation .2. Folding Pathway for Protein. *J. Phys. Soc. Japan*, **44**, 1939-1945.
2. Muñoz, V. and Eaton, W.A. (1999) A simple model for calculating the kinetics of protein folding from three-dimensional structures. *Proc. Natl. Acad. Sci. U.S.A.*, **96**, 11311-11316.
3. Naganathan, A.N. (2012) Predictions from an Ising-like Statistical Mechanical Model on the Dynamic and Thermodynamic Effects of Protein Surface Electrostatics. *J. Chem. Theory Comput.*, **8**, 4646-4656.
4. Taketomi, H., Ueda, Y. and Go, N. (1975) Studies on Protein Folding, Unfolding and Fluctuations by Computer-Simulation .1. Effect of Specific Amino-Acid Sequence Represented by Specific Inter-Unit Interactions. *Inter. J. Prot. Pep. Res.*, **7**, 445-459.
5. Naganathan, A.N. (2013) A Rapid, Ensemble and Free Energy Based Method for Engineering Protein Stabilities. *J. Phys. Chem. B*, **117**, 4956-4964.
6. Robertson, A.D. and Murphy, K.P. (1997) Protein structure and the energetics of protein stability. *Chem. Rev.*, **97**, 1251-1267.
